# Evolution of the connectivity and indispensability of a transferable gene: the simplicity hypothesis

**DOI:** 10.1186/s12862-022-02091-w

**Published:** 2022-11-30

**Authors:** C. T. Jones, E. Susko, J. P. Bielawski

**Affiliations:** 1 Department of Biochemistry and Molecular Biology, Dalhousie University, Halifax, NS Canada; 2 Department of Mathematics and Statistics, Dalhousie University, Halifax, NS Canada; 3 Department of Biology and Department of Mathematics and Statistics, Dalhousie University, Halifax, NS Canada

**Keywords:** Horizontal gene transfer, Connectivity, Indispensability, Gene-host coevolution, Constructive neutral evolution, The simplicity hypothesis

## Abstract

**Background:**

The number of interactions between a transferable gene or its protein product and genes or gene products native to its microbial host is referred to as connectivity. Such interactions impact the tendency of the gene to be retained by evolution following horizontal gene transfer (HGT) into a microbial population. The complexity hypothesis posits that the protein product of a transferable gene with lower connectivity is more likely to function in a way that is beneficial to a new microbial host compared to the protein product of a transferable gene with higher connectivity. A gene with lower connectivity is consequently more likely to be fixed in any microbial population it enters by HGT. The more recently proposed simplicity hypothesis posits that the connectivity of a transferable gene might increase over time within any single microbial population due to gene-host coevolution, but that differential rates of colonization of microbial populations by HGT in accordance with differences in connectivity might act to counter this and even reduce connectivity over time, comprising an evolutionary trade-off.

**Results:**

We present a theoretical model that can be used to predict the conditions under which gene-host coevolution might increase or decrease the connectivity of a transferable gene over time. We show that the opportunity to enter new microbial populations by HGT can cause the connectivity of a transferable gene to evolve toward lower values, particularly in an environment that is unstable with respect to the function of the gene’s protein product. We also show that a lack of such opportunity in a stable environment can cause the connectivity of a transferable gene to evolve toward higher values.

**Conclusion:**

Our theoretical model suggests that the connectivity of a transferable gene can change over time toward higher values corresponding to a more sessile state of lower transferability or lower values corresponding to a more itinerant state of higher transferability, depending on the ecological milieu in which the gene exists. We note, however, that a better understanding of gene-host coevolutionary dynamics in natural microbial systems is required before any further conclusions about the veracity of the simplicity hypothesis can be drawn.

**Supplementary Information:**

The online version contains supplementary material available at 10.1186/s12862-022-02091-w.

## Background

Horizontal gene transfer (HGT) has played an important role in the evolution of archaea, bacteria, and even unicellular eukaryotes [6]. The degree of dispersal of genes acquired by HGT varies and depends in part on features of the transferred material [10, 18, 42]. Empirical studies have suggested that informational genes that code for products involved in the formation of modular supramolecular complexes are less likely to be transferred into new lineages compared to operational genes that carry out more basic functions [20, 21]. This led to the formulation of the “complexity hypothesis”, which in its initial form posited that an informational gene is less likely to be fixed following HGT compared to an operational gene because any new microbial lineage it enters is less likely to contain the protein partners in the configurations it requires for appropriate complex formation [20].

The number of interactions between a transferable gene or its protein product and host genes or gene products is referred to as its connectivity [10]. It has been suggested that it is not only protein–protein interactions associated with complex formation but all forms of interaction that impact transferability or the tendency of a gene to be successfully transferred and retained by evolution [10, 28, 42]. The complexity hypothesis, more generally construed, therefore states that the protein product of a transferable gene with lower connectivity is more likely to function in a way that is beneficial to a new host cell compared to the protein product of a transferable gene with higher connectivity [1]. A transferable gene with lower connectivity is consequently more likely to be fixed by selection or drift in any new microbial population it enters by HGT.

In a recent paper Novick and Doolittle [32] reasoned that the connectivity of a transferable gene might increase over time within any single microbial population by gene-host coevolution. At the same time, the gene might become less vulnerable to deletion in an environment in which it confers no fitness advantage by becoming more indispensable to the viability of its current microbial host. They further proposed that the connectivity of a transferable gene might decrease over time, or at least change in a way that counters the increase in connectivity caused by gene-host coevolution, if given opportunity to colonize naïve microbial populations (i.e., those not yet exposed to the transferable gene). In this way, the “simplicity hypothesis” (ibid) envisions evolution toward a more “sessile” state under which the transferable gene is more resistant to gene loss but also less transferable, or a more “itinerant” state under which the gene is less resistant to gene loss but more transferable, each outcome depending on the prevailing ecological conditions.

In this article we present a simple model designed to test the “theoretical veracity” of the simplicity hypothesis. Most models of HGT consider processes that occur within a single microbial population and make no reference to connectivity (e.g., [30, 33, 40]). Here we take a novel approach based on three assumptions. First, we assume that coevolutionary processes can change both the connectivity and, independently, the indispensability of a transferable gene over macroevolutionary time. Second, we imagine a collective of spatially segregated microbial populations, not all necessarily of the same strain or species, some of which host the transferable gene and some of which are naïve. Third, we admit an environment with two states, one in which the transferable gene provides a fitness advantage to its microbial host (the selective state) and one in which it does not (the neutral state). Using simulations, we explore the conditions under which the transferable gene might evolve to become more sessile or more itinerant over macroevolutionary time scales.

### Model overview

Whether a genic novelty is fixed or eliminated within a microbial population depends on several factors. These include the selection coefficient for a cell with that novelty compared to a cell without it and the effective size of the microbial population. In the case where the novelty is a transferable gene, there is also the rate at which naïve cells acquire the gene from other cells in the same population by HGT. A variant of Kimura’s diffusion approximation [22] that accounts for these factors has been proposed [40]. We nevertheless choose to consign such factors to the background. We instead focus our attention on processes that occur at the level of a metapopulation of transferable genes. These include the formation of new populations of the transferable gene via HGT and the elimination of populations of the transferable gene by gene loss while the environment is in the neutral state. Similarly, although the mechanisms by which a cell can acquire foreign genetic material by HGT are complex and varied (e.g., transformation, transduction, and conjugation, see Arnold et al. [2] for a comprehensive review), the impact of the differences between these are not considered.

Let T represent a transferable gene and P its protein product. The **indispensability** of T is defined to be the degree to which the viability of a microbial host population depends on the presence of T when the environment is in the neutral state. This is represented by ${y}_{i}\in \{\mathrm{0,1},2,\dots \}$, a property of the microbial host population corresponding to the number of dependencies on T the host has accumulated via gene-host coevolution (see Table 1 for a list model parameters). The **connectivity** of T is defined to be the degree to which P of T requires the specific cellular environment provided by its current microbial host to function in a way that provides a fitness advantage to host cells when the environment is in the selective state. This is represented by ${z}_{i}\in \{\mathrm{0,1},2,\dots \}$, a property of T itself.

**Table 1:** Description of model parameters

Parameter	Qualitative description	Default values and units
$\left({y}_{i}, {z}_{i}\right)$	The indispensability ($y$) and connectivity ($z$) of the ${i}^{th}$ population of transferable genes	Non-negative integers
$\Delta {x}_{i}$	The realized change in ${y}_{i}$ or ${z}_{i}$ over one ancestor–descendant mapping due to gene-host coevolution	$-1, 0, 1$
$P\left(+1\right)$	The probability that $\Delta {x}_{i}=+1$	${10}^{-3}$ per mapping
$P\left(-1\right)$	The probability that $\Delta {x}_{i}=-1$	${10}^{-5}$ per mapping
$E(\Delta {x}_{i})$	The expected value of $\Delta {x}_{i}$	Expected change per mapping
$N$	The number of populations in the metapopulation of transferable genes	Non-negative integer
${N}_{max}$	The maximum number of populations in the metapopulation of transferable genes	${10}^{4}$
${\beta }_{N}$	The expected number of naïve microbial populations a population of transferable genes will enter by HGT over one ancestor–descendant mapping	A Poisson random variable with mean ${\beta }_{N}=\beta \left(1-N{/N}_{max}\right)$ per mapping $\beta \in \left\{0.06, 0.08\right\}$
$\delta$	The probability that a microbial host population will be temporarily subjected to the neutral environmental state at some point over one ancestor–descendant mapping	$0.01$ per mapping
${p}_{D}\left({y}_{i}\right)$	The probability that a population of T will suffer death by gene loss in the neutral environmental state	$\mathrm{exp}\left(-{y}_{i}s\right)\in \left[\mathrm{0,1}\right], s=0.20$
${p}_{B}\left({z}_{i}\right)$	The probability that a population of T will reproduce following HGT into a naïve microbial population	$\mathrm{exp}\left(-{z}_{i}s\right)\in \left[\mathrm{0,1}\right], s=0.20$

The copies of a transferable gene that reside within any given microbial host population will be construed as a population of transferable genes that can undergo processes of birth and death. Birth occurs when copies of T drawn from one population of transferable genes successfully enter and become fixed within a naïve microbial population. This process is impacted by the connectivity of T (a property intrinsic to T) as well as the level opportunity to colonize naïve microbial populations by HGT (a property of the ecological milieu). Death occurs when T is deleted from a microbial host cell and the descendants of that cell are subsequently fixed in the microbial population. Death by gene loss is impacted by the indispensability of T to its current microbial host (a property intrinsic to the microbial host) and by whether the state of the environment is selective or neutral (a property of the ecological milieu). Hence, the evolutionary trajectory of T is assumed to depend on both gene-host coevolution and the ecological conditions under which host cells exist.

We make the following additional assumptions about the process and impact of gene-host coevolution. First, the indispensability ${y}_{i}$ of the ${i}^{th}$ microbial host population and the connectivity ${z}_{i}$ of the corresponding population of transferable genes are assumed to change over time, each independently of the other, via gene-host coevolution. An increase in the indispensability of T is assumed to be accompanied by a reduction in the probability that the population of transferable genes will be eliminated from its microbial host population by gene loss while the environment is in the neutral state. An increase in the connectivity of T is assumed to be accompanied by a decrease in the probability that the population of transferable genes will successfully colonize the next naïve microbial population it enters by HGT. The dependencies accumulated by T while it resides in its current host population correspond to changes in the gene sequence itself. The connectivity of T is therefore assumed to be carried along with it when it enters a naïve microbial population. In contrast, the dependencies on T accumulated by a microbial host population are assumed to be absent in microbial populations not yet exposed to the transferrable gene. The indispensability of T is therefore set to zero following HGT into a naïve microbial population.

## Results

We use the Price equation [16, 17, 35] to construct a theoretical expression for the change in the mean character state $\left(\overline{y },\overline{z }\right)$ of a collection of ancestral populations of T when it is mapped forward some billions of microbial generations onto a collection of descendant populations of T (i.e., over one “ancestor–descendant” mapping). The mapping (Eqs. 1, 2, 3 in Methods) provides conceptual clarity by separating evolutionary processes that occur at the level of the metapopulation of transferable genes based on the expected rates of birth and death of populations of T from coevolutionary processes that occur within individual microbial host populations based in the expected change in the indispensability and connectivity of populations of T. The Price equation is deterministic, as it does not account for the stochastic nature of the modelled processes. Hence, we use stochastic computer simulations to investigate the impact of random processes on the trajectory of the mean character state $\left(\overline{y },\overline{z }\right)$.

### Gene-host coevolution under the deterministic model

**Fig. 1:**
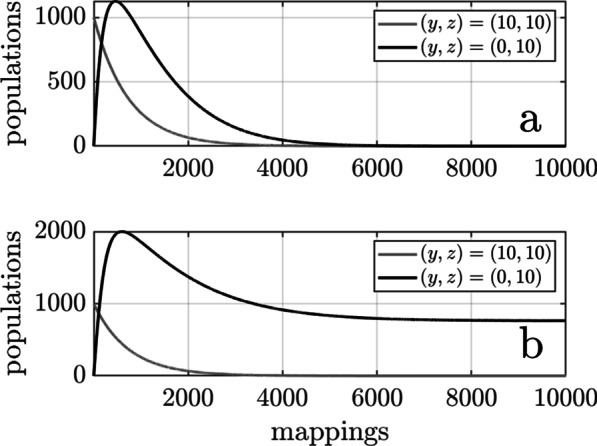
The fate of a transferable gene as a function of $\beta$ under the deterministic model: Whether a metapopulation of transferable genes persists or goes extinct depends in part on the level of opportunity ($\beta$) to colonize naïve microbial populations by HGT. The two panels show the fate of $1000$ populations of the transferable gene, each starting with indispensability and connectivity $\left(y,z\right)=\left(\mathrm{10,10}\right)$, after ${10}^{4}$ ancestor–descendant mappings in the absence of mutations in the indispensability and connectivity of T. In both panels the original $1000$ populations of T were eliminated after 5100 mappings. The fate of their offspring populations with $\left(y,z\right)=\left(\mathrm{0,10}\right)$ then depended on $\beta$. **a** When $\beta =0.06$ the offspring populations went extinct after 7120 mappings. **b** When $\beta =0.08$ they persisted at an equilibrium of approximately 800 populations. In both cases the environment was predominately selective, the probability of exposure to the neutral state having been set to $\delta =0.01$ per ancestor–descendant mapping. Extinction would be more rapid for larger values of $\delta$

What does our model reveal about the possibility of the simplicity hypothesis in nature? Let us first examine the fate of a homogeneous metapopulation of transferable genes in the absence of mutations in the indispensability or connectivity of T. Suppose, for example, that the character state of all populations of T is $\left(y,z\right)=\left(10, 10\right)$ and that the probability that a microbial host population will be subjected to the neutral environmental state (represented by “delta” for “death”, $\updelta \in \left[\mathrm{0,1}\right]$) is only $\updelta =0.01$ per ancestor–descendant mapping, meaning that the environment is predominantly selective. Using hypothetical values for all model parameters (see Methods), each population is expected to persist for approximately $740$ ancestor–descendant mappings before suffering death by gene loss, and an initial set of one thousand such populations is expected to be eliminated after approximately $5100$ mappings. The metapopulation of transferable genes can therefore avoid extinction only by generating new populations of T by HGT. However, a population with $\left(y,z\right)=\left(0, 10\right)$ generated by HGT is expected to persist for only $99$ mappings before death. Extinction is therefore inevitable unless the rate at which $\left(y,z\right)=\left(0, 10\right)$ populations multiply by HGT is great enough to compensate for their short lifespan. The rate of multiplication is determined by the level of opportunity to colonize naïve microbial populations (represented by “beta” for “births”, $\beta \ge 0$). When that opportunity is smaller ($\beta =0.06$), the metapopulation of transferable genes with $\left(y,z\right)=\left(0, 10\right)$ is expected to persist for approximately $7120$ mappings before going extinct (Fig. 1a). When the opportunity is slightly larger ($\beta =0.08$), the metapopulation of transferable genes with $\left(y,z\right)=\left(0, 10\right)$ will persist indefinitely (Fig. 1b). In either case, multiplication by HGT lowers the indispensability of T, making it vulnerable to death by gene loss in environments in which $\updelta$ is closer to one.

Now consider a metapopulation in which the character state of all populations of T is $\left({y}_{1},{z}_{1}\right)$ apart from one population with the same indispensability but with lower connectivity $\left({y}_{2},{z}_{2}\right)=\left({y}_{1},{z}_{1}-1\right)$. The former population plays the role of a wild type and the latter the role of a mutant.^1^ It can therefore be asked whether the mutant will eventually be fixed (i.e., remain as the only population variant in the metapopulation of transferable genes). Let us again set aside the possibility of gene-host coevolution so that no further mutant populations can arise. Since HGT is assumed to result in the loss of indispensability, the wild type and mutant populations can only generate descendants by HGT with character state $\left(0,{z}_{1}\right)$ and $\left(0,{z}_{2}\right)$, respectively. And since the probability of death by gene loss is positive, the original wildtype populations of T and the single mutant population of T will eventually be eliminated. As to the fate of the new population variants $\left(0,{z}_{1}\right)$ and $\left(0,{z}_{2}\right)$, it is evident that there will eventually be more of the latter. For whereas both variants suffer the same probability of death, both having indispensability $y=0$, the mutant population will generate more descendants due to its lower connectivity. This does not mean that the mutant will eventually be fixed, however. Fixation will only occur if the $\left(0,{z}_{2}\right)$ population variant can avoid extinction, or in other words only if the expected number of births by HGT exceeds the expected probability of death. This defines the condition under which a chance reduction in connectivity in one population of T can be amplified by the opportunity to colonize naïve populations by HGT afforded by larger $\beta$.

Finally, let us consider a metapopulation in which the character state is $\left({y}_{1},{z}_{1}\right)$ in some populations and $\left({y}_{2},{z}_{2}\right)=\left({y}_{1}+1,{z}_{1}\right)$ in others. If the baseline rate at which T enters naïve populations by HGT is very low (i.e., if $\beta \approx 0$), then all populations of T will eventually be eliminated and the metapopulation of transferable genes will go extinct. However, the $\left({y}_{2},{z}_{2}\right)$ population variant will persist longer due to its greater indispensability. The proportion of $\left({y}_{2},{z}_{2}\right)$ populations of T will consequently increase over time despite the dwindling number of both variants. Let us further suppose that the probability of exposure to the neutral environmental state is very low (i.e., $\delta \approx 0$). Then the extinction of T will take many ancestor–descendant mappings. The longer it takes for extinction to occur, the greater the probability that the indispensability of T will be increased by gene-host coevolution in some populations (i.e., when mutations that change $y$ and $z$ are allowed). Any such increase will extend the time before extinction and increase the probability that the indispensability of T will increase yet again in some populations. Interestingly, this scenario is consistent with the idea that “selection by survival” without reproduction can result in evolutionary change [7, 12, 14, 27]. Under the present model, populations of T cannot reproduce in the absence of naïve microbial populations. But any one population of T can evolve to become more indispensable to its current host, making it more likely that it will persist indefinitely.

### Gene-host coevolution under the stochastic model

**Fig. 2:**
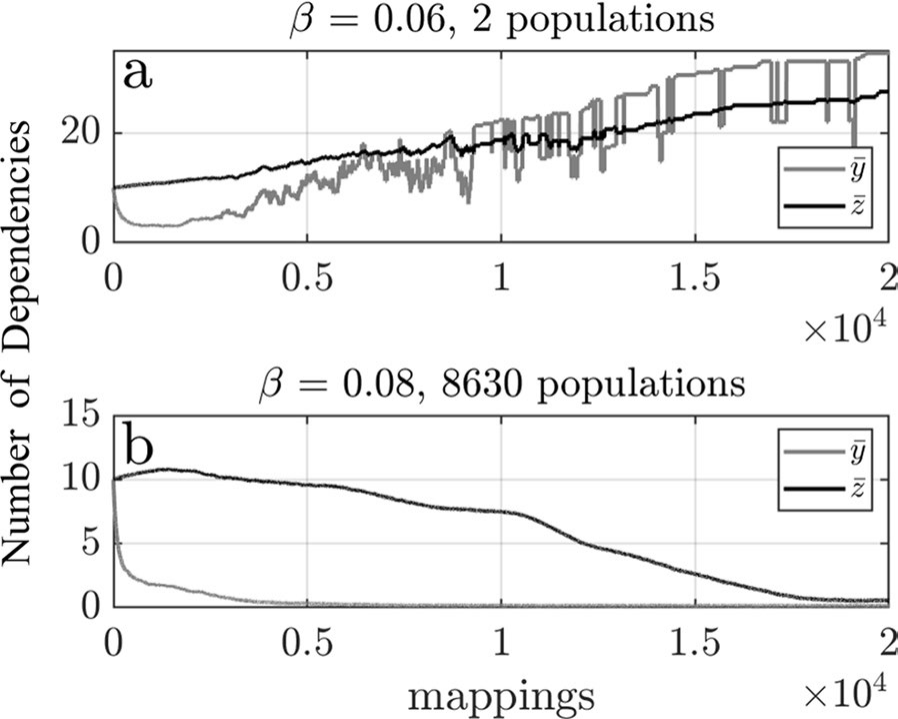
The fate of a transferable gene as a function of $\beta$ under the stochastic model: The metapopulation of transferable genes will tend to evolve toward a more sessile or itinerate state depending on the level of opportunity ($\beta$) to colonize naïve microbial populations by HGT. The two panels show the fate of $1000$ populations of the transferable gene, each starting with $\left(y,z\right)=\left(\mathrm{10,10}\right)$, after ${10}^{4}$ ancestor-mappings under the stochastic model. **a **The transferable gene evolved toward a more sessile state with larger mean indispensability ($\overline{y }$) and mean connectivity ($\overline{z }$) when $\beta =0.06$. This was accompanied by a huge reduction in the number of populations, down to only 2 by the end of the simulation. **b** The transferable gene evolve toward a more itinerant state with smaller mean indispensability ($\overline{y }$) and mean connectivity ($\overline{z }$) when $\beta =0.08$. This was accompanied by a more than eight-fold increase in the size of the metapopulation up to 8630 populations of the transferable gene

Let us now turn to a scenario in which gene-host coevolution does occur, and in which all processes, including birth by HGT, death by gene loss, and change in the indispensability and connectivity of T occur stochastically. We again start with one thousand populations of T with $\left(y,z\right)=\left(10, 10\right)$ but this time allow the possibility of gene-host coevolution with a strong mutational bias toward larger $y$ and $z$. Two evolutionary trajectories generated under the stochastic model are shown in Fig. 2.

A comparison of the outcomes in Figs. 1 and 2 illustrates the difference coevolution can make. When $\beta =0.06$, the transferable gene went extinct in the absence of gene-host coevolution (Fig. 1a). However, T managed to persist in two populations under the stochastic model (Fig. 2a). This was due to a gradual increase in the indispensability of T in those few microbial populations in which T managed to persist to the end of the simulation. But note that this came at the expense of a similar increase in connectivity. Hence, the transferable gene persisted to the end of the simulation by evolving toward a more sessile state. When $\beta =0.08$ the transferable gene was maintained in approximately 800 populations in the absence of coevolution (Fig. 1b). This number increased dramatically to close to nine thousand populations when coevolution was allowed (Fig. 2b). The increase was driven by chance reductions in the connectivity of some populations of T, which allowed more rapid dispersal into naïve microbial populations. The result was a gradual reduction in the mean connectivity of T but at the expense of a decrease in its mean indispensability. Hence, the transferable gene capitalized on the opportunity to colonize naïve microbial populations by evolving toward a more itinerant state at the expense of becoming more vulnerable to death by gene loss.

**Fig. 3:**
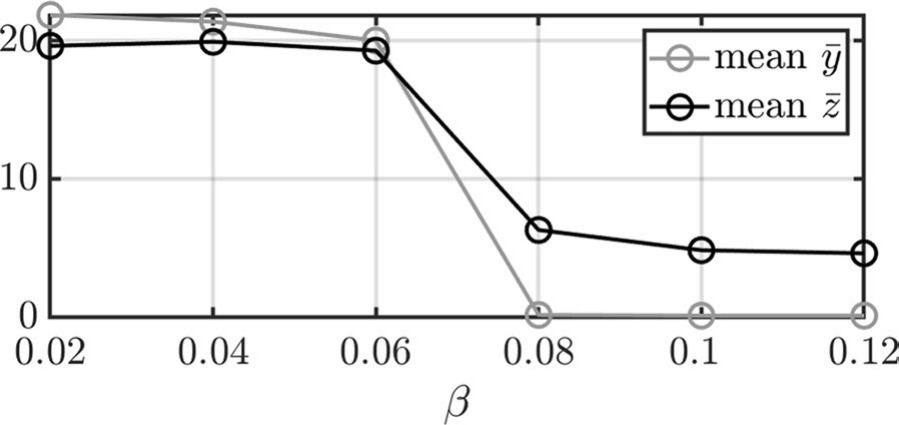
Transition from sessile to itinerant as a function of $\beta$ under the stochastic model: The direction of the evolutionary trajectory of the metapopulation of transferable genes switches from more sessile to more itinerate as the opportunity to colonize naïve microbial populations increases past a threshold value $\beta >0.06$. Fifty stochastic simulations were conducted with each value of $\beta$ considered starting with ${10}^{4}$ populations of the transferable gene with $\left(y,z\right)=\left(10, 10\right)$. Each circle shows a mean value taken across all fifty simulations. The evolutionary outcome changed from sessile (corresponding to higher mean $\overline{y }$ and $\overline{z }$) to itinerant (corresponding to lower mean $\overline{y }$ and $\overline{z }$) somewhere between $\beta =0.06$ and $\beta =0.08$. This demonstrates how a combination of gene-host coevolution within populations and either differential rates of persistence or differential rates of multiplication by HGT can cause the transferable gene to evolve to become more sessile or more itinerant, respectively, depending on the level of opportunity to colonize naïve microbial populations by HGT

Our analysis of Fig. 2 suggests that the evolutionary outcome under the parameter settings considered turns from sessile to itinerant somewhere between $\beta =0.06$ and $\beta =0.08$. To explore this, simulations were conducted with different values for $\beta \in \left[0.06, 0.08\right]$. Figure 3 shows that the mean indispensability $\overline{y }$ was approximately $20$ by the end of most simulations when $\beta \le 0.06$ but evolved to a value less than the initial value $y=10$ when $\beta \ge 0.08$. The mean $\overline{z }$ exhibits a similar transition. This illustrates how the mean indispensability and mean connectivity of a metapopulation of transferable genes can be correlated within a shared ecological milieu even when they change according to independent evolutionary dynamics. It also shows how a transferable gene can evolve toward a more sessile or more itinerant state under the assumed model depending on ecological conditions that determine the level of opportunity to colonize naïve microbial populations.

These simple simulations demonstrate how gene-host co-evolution combined with the prevailing ecological conditions might cause the indispensability and connectivity of a transferable gene to change over macroevolutionary time scales. Of course, results will vary depending on the values chosen for model parameters. It is possible to find parameter settings under which T will evolve to be more sessile or more itinerant, as well as settings under which T will go extinct before it can adapt to its current environmental milieu. However, it is difficult to determine what parameter values would be most consistent with what happens in a natural system. Consider, for example, a microbial host population of effective size ${N}_{e}$. If ${\mu }_{0}$ is the rate at which neutral mutations that impact T or host genes arise per host cell per generation, then, according to neutral theory [23], the rate of change in the combined T-plus-host genome by such mutations is:

$$\mathrm{probability\,of\,mutation} \times \mathrm{probability\,of\,fixation}= {\mu }_{0}{N}_{e}\times \frac{1}{{N}_{e}}={\mu }_{0}$$

The rate of change in the indispensability of a transferable gene by neutral processes (e.g., constructive neutral evolution) while it resides in its current microbial host population would be a fraction of this, say ${\mu }_{y}<{\mu }_{0}$. Likewise, for connectivity, ${\mu }_{z}<{\mu }_{0}$. To assess whether a transferable gene might evolve to become more sessile or itinerant by neutral processes, it would be necessary, at the very least, to estimate ${\mu }_{y}$ and ${\mu }_{z}$. To the best of our knowledge, no such estimates exist. A similar statement can be made for other kinds of mutations that change the state of a transferable gene, such as those that alter T or the host cell in such as way as to increase host fitness.

## Discussion

The objective of this study was to test the intuition, expressed by Novick & Doolittle [32], that if it were possible for a transferable gene T and its microbial host to coevolve in such a way as to change the indispensability and connectivity of T over macroevolutionary time, then the transferable gene might evolve to become more itinerant or more sessile, depending on the ecological conditions under which it exists. The present theoretical model shows that the opportunity to colonize naïve microbial populations can exert a selective pressure that acts to reduce the mean connectivity and mean indispensability of a metapopulation of transferable genes over time, making the gene more itinerant. On the other hand, a lack of such opportunity combined with a low probability of death by gene loss can result in a kind of “selection by survival” [7, 12, 14, 27] that increases the mean connectivity and mean indispensability of the transferable gene over time, making the gene more sessile. These results are consistent with the simplicity hypothesis.

The veracity of the simplicity hypothesis depends on many factors, however, including the rate at which mutations that alter indispensability and connectivity might arise. We can nevertheless make two predictions predicated on the assumption that that gene-host coevolution occurs at a rate great enough to effect change (e.g., [26]). First, a transferable gene might evolve to become more sessile when there is a low probability of gene loss due to a stable selective environment but also little opportunity to colonize naïve microbial populations. The first condition (analogous to $\delta \approx 0$) ensures that some populations of the transferable gene will reside in their current microbial hosts long enough for the sessile state to evolve, while the second condition (analogous to $\beta \approx 0$) curtails competition with more itinerant variants of T. Second, a transferable gene might evolve to become more itinerant when the opportunity to colonize naïve microbial populations is high enough to compensate for gene loss.^2^

These predictions may be testable. Suppose the protein product P1 of a transferable gene T1 is known to confer the ability to metabolize a substrate $S$. If the environment in which the gene exists is characterized by low levels of growth-limiting nutrients (e.g., iron) so that HGT into naïve microbial populations is rare ($\beta \approx 0$) but also by efficient cross-feeding so that the supply of $S$ is continuous ($\delta =0$), then our model predicts that T might evolve to become more sessile. Such genes may be common in microbes that exist in oligotrophic environments such as the open ocean, deep subsurface soils, or under the polar ice caps. For comparison, suppose P2 of T2 confers the ability to resist an antimicrobial substance $A$. If productivity is high due to nutrient abundance so that HGT into naïve microbial populations is common ($\beta >0$) but exposure to $A$ is intermittent ($\delta >0$), then our model predicts that T2 might evolve to become more itinerant. Genes of this type might be found in various pathogens that have been episodically exposed to antimicrobial substances. Our theoretical results might therefore be tested by comparing the mean indispensability and mean connectivity of genes of type T1 with that of genes of type T2 if such genes can be identified. Our predictions might also be tested by comparing a transferrable gene found in some species having broad niche breadth with paralogs found in species having narrow niche breadth. Such genes should be more itinerant in the former sort of species and more sessile in the latter.

Gene acquired by HGT can be identified using parametric methods based on measurable properties of genome segments that tend to exhibit low variability within genomes and high variability between genomes (e.g., GC content, nucleotide composition, oligonucleotide frequencies [3]), phylogenetic methods that compare gene trees with species trees to identify inconsistencies that might be explained by HGT [36], and methods based on models of gene gain/loss [9, 44]. The number of species in which a transferable gene is found might be used as a proxy for the degree to which the gene is itinerant. This can be compared to the connectivity of such genes, which can be estimated using the Database of Interacting Proteins (DIP, [43]) or the Search Tool for the Retrieval of Interacting Proteins (STRING, [39]). It would therefore seem to be possible to compare the connectivity and itinerance of genes of type T1 and T2 using existing data.

Indispensability, however, is host-specific and might require in vitro experiments to estimate, for example by assaying fitness following gene loss in a neutral environment.^3^ Moreover, there are other factors that can impact itinerance as a proxy for transferability, as evidenced by the fact that genes with the same connectivity can vary widely in the number of microbial lineages in which they are found (e.g., Fig. 1 in [10]). Factors considered in other empirical studies include gene size [11], gene duplicability [42], gene “friendliness” [18], and the level of gene expression [34]. Transferability also depends on the selection coefficient for a cell with the gene compared to one without, the effective size of the microbial population, the rate of HGT within that population [40], and the availability of naïve microbial populations to colonize by HGT. Designing an empirical study to test the simplicity hypothesis may therefore be possible but would likely pose a considerable challenge.

## Conclusion

Our theoretical results support the simplicity hypothesis as well as the general prediction that transferable genes found in microbes adapted to a specific environment (narrow niche-breadth) will tend to be more sessile, whereas those found in microbes adapted to a wider range of environmental conditions (broad niche-breadth) will tend to be more itinerant. However, further assessment regarding the possibility of the simplicity hypothesis must await an advance in our understanding of gene-host coevolutionary dynamics in natural microbial systems.

## Methods

### The simplicity hypothesis and constructive neutral evolution

Let T represent a transferable gene encoding a single protein product P that confers a fitness advantage to a host cell under specific environmental conditions (e.g., depending on available nutrients, temperature, salinity, pH, the presence of antimicrobial substances, etc.). The copies of a transferable gene that reside within a microbial host population will, for the purpose of our model, be construed as a population of transferable genes or a “population of T”. The milieu of the simplicity hypothesis is a “population of populations” or “metapopulation” [29] of transferable genes. Naïve microbial populations into which T can be transferred are also assumed to exist.

The simplicity hypothesis requires connectivity to vary across populations of T. One possible source of variation, proposed by Novick and Doolittle [32], is constructive neutral evolution (CNE). Broadly speaking, CNE occurs when a mutation in one gene (whether transferable or not) that would be deleterious is rendered neutral or nearly neutral due to a fortuitous or previously selected association with another gene that “pre-supresses” the deleterious effect of that mutation [19]. The fixation of the mutation by drift will result in an increase in the dependency between the two genes, which would subsequently be maintained by purifying selection. In the case of gene duplication, for example, a mutation in one copy that would otherwise reduce the fitness of an organism can be fixed by drift due to the presence of the second copy. The performance of a function that was once carried out by the original copy might thereby come to depend on the existence of both paralogs via a process known as subfunctionalization [15, 38]. See Muñoz-Gómez et al. [31] and citations therein for other examples of genetic features that can be explained by CNE.

In the specific context of a transferable gene, it is assumed that mutations in T that reduce or eliminate the fitness advantage P confers to a host cell can sometimes arise. It is further assumed that the impact of such mutations on P can sometimes be neutralized by the presence of host genes or gene products. The fixation of any such mutation by drift will increase the degree to which the fitness advantage P provides depends on genes or gene products specific to the microbial population that currently hosts the population of T. In this way, CNE can increase the connectivity of T and decrease the probability that P will function in a way that provides a fitness advantage to the next naïve microbial host cell that T enters by HGT. Similarly, it is assumed that mutations in host genes that reduce host fitness can sometimes be neutralized by the presence of T. The fixation of such mutations by drift will increase the degree to which the viability of the host population depends on the presence of T. This will reduce the probability that T will be lost from that host population, as would otherwise be likely in the event of a change in the environment that negates the fitness advantage P provides (e.g., [5, 25]). Any such increase in host dependency is referred to as an increase in the indispensability of T to its current host population.

The preceding demonstrates how, in theory, constructive neutral evolution can gradually increase the indispensability and, independently, the connectivity of a population of transferable genes while it resides within its current microbial host population. CNE can therefore act as a complexity ratchet to produce what Novick and Doolittle [32] call a sessile transferable gene, one unlikely to suffer gene loss from its current microbial host population due to its high indispensability, but also unlikely to be fixed following transfer into a naive microbial population due to its high connectivity. This trend toward greater complexity and the sessile state can be opposed by the opportunity to colonize naïve microbial populations by HGT. The indispensability of T in a newly colonized microbial population is minimal since it takes time for the new host to accumulate dependencies on T. High rates of colonization will therefore reduce the mean indispensability of T across a metapopulation of transferable genes and increase the probability that some populations of T will be eliminated by gene loss. However, the opportunity to colonize also favors the dispersal of variants of T with lower connectivity. The opportunity to disperse by HGT can therefore act as a simplicity ratchet to produce what Novick and Doolittle [32] call an itinerant transferable gene, one quite likely to colonize naïve microbial populations due to its low connectivity, but also unlikely to persist in any one microbial host population for long due its low indispensability.

### Changes in connectivity

A functional module is a group of genes or gene products related by genetic or intracellular interactions [41]. Functional modules are often displayed as a graph with nodes representing genes or their protein products and edges indicating relationships between nodes. In this context, the connectivity of a gene (whether transferable or not) is just the number of edges connecting it to other nodes in the same gene co-expression or protein interaction network [8]. A change in the connectivity of a gene corresponds to a change in the number of such edges. This can occur in several ways, depending on the gene. If the gene codes for a protein that is part of a supramolecular complex, then any change in the number of subunits that make up the complex will change the gene’s connectivity. The evolution of tetrameric hemoglobin from a monomeric ancestral protein provides an example [4]. Connectivity can also be altered by a change in the number of proteins involved in a metabolic, signaling, or regulatory pathway. The transcription factor SIM1, for example, plays several roles in humans, from the development of neurons during embryogenesis to the regulation of functions in the adult form. The STRING [39] database indicates that SIM1 is involved in eight direct protein–protein interactions in humans but only one in *Mus musculus*, suggesting that the connectivity of SIM1 might have changed over macroevolutionary time scales.

Here we posit an additional and, in some ways, more subtle process of change. The protein product P of T needs to fold into a specific stable configuration and may require access to one or more specific binding partners to carry out its selected function (i.e., the function that is beneficial to the host under some environmental conditions). We assume that mutations in T that would cause P to become unstable or unable to carry out its selected function can be pre-suppressed or rendered neutral by the presence of genes or gene products native to the host. The fixation of such mutations by drift would increase the connectivity of the transferable gene, here broadly construed as the degree to which P depends on the specific intracellular milieu provided by its current host to function. We also entertain the possibility that other mutations in T can remedy the need for the suppression of previously fixed mutations, and that these can lead to a reduction in the connectivity of T if fixed, although such reversions are presumably rare (e.g., [19]).

### Changes in indispensability

An essential gene is defined to be one that supports a function that is necessary for reproductive success (e.g., genes required for transcription and translation). Such genes tend to correspond to nodes in functional modules with many edges and so are typically not transferable but rather part of a core genome common to a wide range of strains or species [37]. Interestingly, there is evidence to suggest that the essentiality of a gene is nevertheless mutable and subject to evolutionary processes (ibid). Here we define the indispensability of a transferable gene to be the degree to which the viability of a microbial host population comes to depend on T via a process of gene-host coevolution. By this definition, an indispensable transferable gene is in some ways like an essential gene. However, an increase in the indispensability of T does not necessarily make the transferable gene essential or necessary for reproductive success. Instead, we imagine that a transferable gene can sometimes insinuate itself into the protein networks of its host by CNE in a Rube Goldberg fashion until the cell can no longer survive without it [19]. This can occur if the presence of T acts to pre-suppress the deleterious effects of mutations that arise in host genes. The fixation of such mutations by drift will increase the indispensability of T while it resides in its current microbial host population, making it less likely that the host population will lose T in an environment in which the selected function of P provides no fitness advantage.

### Accounting for gene-host coevolution

Let $P\left(\Delta {x}_{i}\right), {x}_{i}\in \left\{{y}_{i},{z}_{i}\right\}$ represent the probability that a mutation that changes the indispensability $({y}_{i})$ or connectivity ${(z}_{i})$ of T from ${x}_{i}$ to ${x}_{i}+\Delta {x}_{i}$ arises in one copy of the transferable gene and is subsequently fixed in its current microbial host population by drift. Three outcomes are considered, $\Delta {x}_{i}\in \left\{-\mathrm{1,0},+1\right\}$, when ${x}_{i}\ge 1$, and two, $\Delta {x}_{i}\in \left\{0,+1\right\}$, when ${x}_{i}=0.$ In both cases, $\Delta {x}_{i}=0$ indicates that no mutation occurred or that one occurred but was not fixed. It is assumed that change rarely occurs, so $P\left(0\right)\approx 1$, and that mutations are biased to increase both the indispensability and connectivity of T, so $P\left(+1\right)\gg P\left(-1\right)$. This is consistent with the general view that constructive neutral evolution is a rare process that tends to increase complexity over time [31]. The expected change in ${y}_{i}$ and ${z}_{i}$ over one ancestor–descendant mapping is therefore:

1 $$\mathrm{E}\left(\Delta {x}_{i}\right)=\left\{\begin{array}{c} P\left(+1\right)-P\left(-1\right), {x}_{i}\ge 1\\ \frac{P\left(+1\right)}{P\left(0\right)+P\left(+1\right)}, {x}_{i}=0\end{array}\right.$$

The key assumption of our model is that the indispensability and connectivity of a transferable gene can independently increase or decrease over time via gene-host coevolution. Although CNE provides a plausible mechanism for change [32], the assumption that change occurs by CNE alone is not crucial. In other words, $P\left(\Delta {x}_{i}\right)$ can be interpreted as the probability of change due to all evolutionary processes that might impact the state $\left({y}_{i},{z}_{i}\right)$ of a population of transferable genes, including but not necessarily limited to CNE.

### The fitness of an ancestral population of transferable genes

The loss of a transferable gene from its current microbial host population can be construed as the death of a population of T. Likewise, colonization of a naïve microbial population by HGT can be construed as the birth of a new population of T. It is therefore possible to treat a population of transferable genes as an individual unit that can be assigned fitness in the form of the number of descendant populations it generates. Fitness, once defined, can then be used to map an ancestral metapopulation of transferable genes onto a descendant metapopulation.

The fitness advantage T confers to a host cell is a function of the state of the environment in which its host population resides. This is assumed to vary across microbial populations and over time. Whether the state of the environment changes in such a way as to negate the fitness P confers (i.e., due to a shift to a neutral environmental state) is determined by a Bernoulli random variable with expected value $\delta \in [\mathrm{0,1}]$. Whether an ancestral population of T will suffer death by gene loss following a temporary switch to the neutral environment is assumed to be a Bernoulli random variable with expected value ${p}_{D}\left({y}_{i}\right)$, a function of indispensability. The expected probability of death by gene loss over one ancestor–descendant mapping is therefore the product ${\delta p}_{D}\left({y}_{i}\right)$. It follows that the probability that an ancestral population of transferable genes will persist into the descendant metapopulation by evading death is ${w}_{i}^{p}=1-{\delta p}_{D}\left({y}_{i}\right)$ (superscript “p” for “persistence”).

The number of naïve microbial populations an ancestral T enters by HGT is assumed to be a Poisson random variable with expected value ${\beta }_{N}=\beta \left(1-N{/N}_{max}\right)$, where $N$ is the current number of ancestral populations of T and ${N}_{max}$ is an upper bound placed on the size of the metapopulation of transferable genes (i.e., the maximum number of populations of T it may contain). Whether T is fixed following HGT is assumed to be a Bernoulli random variable with expected value ${p}_{B}\left({z}_{i}\right)$, a function of connectivity. The expected number of new populations of the transferable gene generated by an ancestral population over one ancestor–descendant mapping is therefore ${w}_{i}^{m}={\beta }_{N}{p}_{B}\left({z}_{i}\right)$ (superscript “m” for “multiplication”).

The expected fitnessof an ancestral population of transferable genes as a function of its indispensability and connectivity is just the sum of the contributions made by persistence and multiplication, ${w}_{i}={w}_{i}^{p}+{w}_{i}^{m}$. The specification of fitness is complete once the functional forms for the probabilities ${p}_{D}\left({y}_{i}\right)$ and ${p}_{B}\left({z}_{i}\right)$ have been chosen. It is not clear what forms these probabilities should take to best reflect what might occur in nature apart from the plausible assumption that both are decreasing functions. For the sake of simplicity, we assume a common exponential form:

2 $${w}_{i}={w}_{i}^{p}+{w}_{i}^{m}=1-\delta \mathrm{exp}\left(-{y}_{i}s\right)+{\beta }_{N}\mathrm{exp}\left(-{z}_{i}s\right)$$

**Fig. 4:**
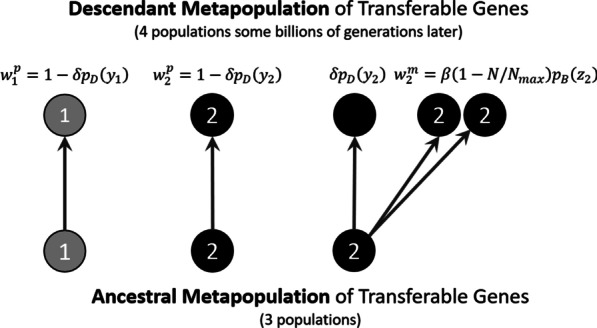
Birth and death process for populations of transferable genes: Circles represent microbial populations, each of which hosts a population of transferable genes with indispensability and connectivity $\left({y}_{1},{z}_{1}\right)$ for transferable gene type ${T}_{1}$ and $\left({y}_{2},{z}_{2}\right)$ for transferable gene type ${T}_{2}$. It is assumed that ${y}_{1}>{y}_{2}$ and${z}_{1}>{z}_{2}$, meaning that ${T}_{1}$ is more sessile and ${T}_{2}$ is more transient. The ancestral metapopulation contained one population of type ${T}_{1}$ and two populations of type${T}_{2}$. In this imaginary scenario, the type ${T}_{1}$ population, being more sessile, survived into the descendant metapopulation of transferable genes, its probability of doing so being ${w}_{1}^{p}=1-{\delta p}_{D}\left({y}_{1}\right)$. It did not produce any new populations, however. One population of type ${T}_{2}$ survived into the descendant metapopulation (probability${w}_{2}^{p}=1-{\delta p}_{D}\left({y}_{2}\right)$) but failed to multiply by HGT. The other type ${T}_{2}$ population was eliminated by gene loss (probability ${\delta p}_{D}\left({y}_{2}\right)$), but managed to generate two new populations before doing so, the expected number of such new populations being ${w}_{2}^{m}=\beta \left(1-N{/N}_{max}\right){p}_{B}\left({z}_{2}\right)$. Note that one ancestor–descendant mapping corresponds to some billions of generations, which is assumed to be long enough for genetic novelties generated by mutation or HGT to be fixed or eliminated

The scaling parameter $s$ controls the rate at which each exponential function approaches its horizontal asymptote at zero. This was set to $s=0.20$ to simulate a relatively slow approach, with an e-fold decrease in probability when ${y}_{i}$ or ${z}_{i}=5$. To restate, $\mathrm{exp}\left(-{y}_{i}s\right)$ models the probability that, following a temporary shift to the neutral environmental state, T is lost from one cell and the lineage of cells without T is subsequently fixed by drift. This is equated to the death of an ancestral population of T over one ancestor–descendant mapping. And $\mathrm{exp}\left(-{z}_{i}s\right)$ models the probability that a lineage of cells with T in an otherwise naïve microbial population reaches fixation. This is equated to the birth of a descendant population of T. The number of microbial generations separating an ancestral metapopulation from its descendant metapopulation is assumed to be more than sufficient for these within-population processes to reach completion (e.g., billions of microbial generations). See Fig. 4 for a depiction of these birth and death processes.

**Fig. 5:**
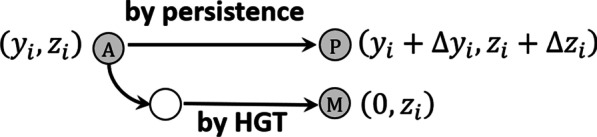
Each gray circle represents a population of transferable genes that resides within a microbial host population. The white circle represents a naïve microbial population, one that has not yet acquired the transferable gene. An ancestral population (A) with character state $\left({y}_{i},{z}_{i}\right)$ can contribute to a descendant metapopulation by avoiding gene loss (by persistence, P) or by colonizing naïve microbial populations by HGT (by multiplication, M). A population generated by persistence inherits its indispensability and connectivity from its ancestor subject to change due to gene-host coevolution, $\left({y}_{i}+\Delta {y}_{i} ,{z}_{i}+\Delta {z}_{i}\right)$. The character state of a population generated by multiplication is $\left(0,{z}_{i}\right)$. In this case indispensability is set to $y=0$ because dependencies accumulated by the ancestral host population are assumed to be absent in the naïve microbial population. The connectivity of T remains ${z}_{i}$ due to the simplifying assumption that gene-host coevolution does not occur until the transferable gene has been fixed following HGT

### The character state of a descendant population of transferable genes

The indispensability and connectivity of a descendant population of transferable genes generated by persistence are inherited, in a manner of speaking, from its ancestral population subject to transmission bias due to gene-host coevolution (Eq. 1). The descendant character state is therefore $\left({y}_{i}+\Delta {y}_{i} ,{z}_{i}+\Delta {z}_{i}\right)$ where $\left({y}_{i},{z}_{i}\right)$ is the state of the ancestral population of T and $\Delta {y}_{i}$ and $\Delta {z}_{i}$ represent any change that might be realized during one ancestor–descendant mapping. The state of a descendant population produced by HGT, by contrast, is $\left(0,{z}_{i}\right)$. Indispensability is set to zero because of the assumption that the dependencies accumulated by an ancestral host population are absent in the naïve microbial population. Connectivity is preserved due to the additional simplifying assumption that gene-host coevolution does not occur until T is fixed in any naïve microbial population it enters. The difference between the two kinds of descendant populations is illustrated in Fig. 5.

### The Price equation

We use the Price equation [16, 17, 35] to write an expression for the change in the mean character state of T in a metapopulation of transferable genes over one ancestor–descendant mapping. Of primary interest is to identify conditions under which the mean connectivity of T will decrease. If ${q}_{i}$ is the proportion of ancestral populations with $\left({y}_{i},{z}_{i}\right)$, then the change in the mean connectivity of T over one mapping is (see Appendix):

3 $$\Delta \overline{z }=\frac{1}{\overline{w}}{\sum }_{i}{q}_{i}\left({w}_{i}^{m}-\overline{w }\right){z}_{i}+\frac{1}{\overline{w}}{\sum }_{i}{q}_{i}{w}_{i}^{p}\left({z}_{i}+\mathrm{E}\left(\Delta {z}_{i}\right)\right)$$

The first sum accounts for differences in the number of descendant populations that each ancestral population of T generates by HGT. Fitness ${w}_{i}^{m}$ and connectivity ${z}_{i}$ are negatively correlated, so this sum is interpreted as the effect of selection that favors populations of T with lower connectivity. The second sum accounts for the expected change in the connectivity of T due to gene-host coevolution within each ancestral population that persists into the descendant metapopulation (see Fig. 5). The expectation $\mathrm{E}\left(\Delta {z}_{i}\right)>0$ is biased toward greater connectivity, so this sum is interpreted as the effect of an evolutionary complexity ratchet. The first sum will tend to decrease the mean connectivity of T in the metapopulation over one ancestral-descendant mapping provided $\mathrm{var}\left({z}_{i}\right)>0$. The second sum will tend to increase the mean connectivity of T over one mapping due to the assumed evolutionary bias toward greater complexity. The direction of change in the mean connectivity of T will therefore depend on the relative size of these two sums, or equivalently, on the tradeoff between multiplication and persistence.

It is important to note that fitness in the Price equation is a realized value, which in our model is equated to an expectation (Eq. 2). The transmission bias is likewise equated to an expected value (Eq. 1). It follows that Eq. 3 is deterministic. It is nevertheless possible to account for stochastic variation in birth, death, and transmission bias by simulating these as random processes, and to use simulations to explore the conditions under which the transferable gene might evolve to become more sessile or more itinerant. See Additional file 1 for details.

### Supplementary Information

**Additional file 1.** Deterministic calculations (Fig. 2). Stochastic simulations (Fig. 3)

## Figures and Tables

**Fig. 6:**
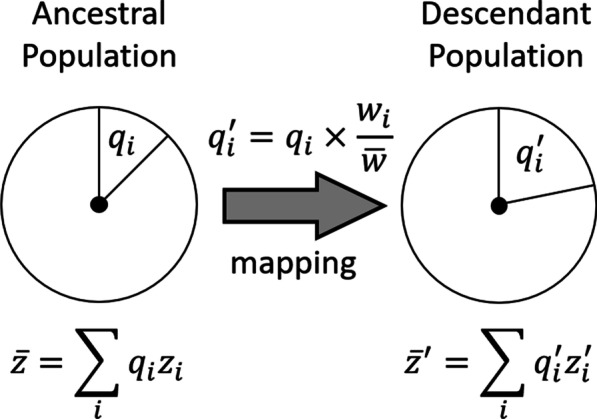
The Price equation can be used to map an ancestral population onto a descendant population over an arbitrary time span in generations. Here ${q}_{i}$ represents the relative frequency of individuals with character state ${z}_{i}$ and fitness ${w}_{i}$ that comprise the ${i}^{th}$ ancestral subpopulation. Its counterpart ${q}_{i}^{^{\prime}}$ is the relative frequency of all individuals that descend from the ${i}^{th}$ ancestral subpopulation. The average character of this descendant subpopulation is ${z}_{i}^{^{\prime}}={z}_{i}+\Delta {z}_{i}$, where $\Delta {z}_{i}$ accounts for the expected change in character state due to transmission bias caused by processes such as mutation and recombination. Change in the mean character state $\Delta \overline{z }={\overline{z} }^{^{\prime}}-\overline{z }$ is a function of differences in the fitness of the ancestral subpopulations and the fidelity with which an ancestral character state is transmitted

## Data Availability

All simulations and calculations were implemented in MATLAB version R2021a under license number 861043 for academic use using custom scripts. Scripts are available on GitHub, https://doi.org/10.5281/zenodo.7194737

## Appendix

### The price equation

See Fig. 6

The Price equation provides a simple way to calculate the change in the mean value of a character state (a phenotype or genotype) in a population of individuals over one ancestor–descendant mapping spanning any number of generations (Fig. 6). The ancestral population consists of some number of individuals $M$ partitioned into subpopulations of size ${m}_{i}$ with the same character state ${z}_{i}$. Each character state corresponds to a fitness ${w}_{i}$ that represents the number of descendants each individual ancestor will produce over the ancestor–descendant mapping. Hence, the ${i}{th}$ ancestral subpopulation will contribute ${m}_{i}\times {w}_{i}$ individuals to the descendant population. The relative frequency of these descendants is:

$${q}_{i}^{^{\prime}}={q}_{i}\times \frac{{w}_{i}}{\overline{w}} \mathrm{where }\,{ q }_{i}=\frac{{m}_{i}}{M}\mathrm\,{and} \,\overline{w }={\sum }_{i}{q}_{i}{w}_{i}$$

The change in relative frequency $\Delta {q}_{i}={q}_{i}^{^{\prime}}-{q}_{i}$ will be positive for subpopulations with greater than average fitness and negative for subpopulations with less than average fitness. This represents the effect of selection. The descendant population will be larger than the ancestral population when the mean fitness $\overline{w }$ is greater than one, and smaller when $\overline{w }<1$.

The mean character state of all descendants generated by an ancestral subpopulation is represented by ${z}_{i}^{^{\prime}}$. The difference $\Delta {z}_{i}={z}_{i}^{^{\prime}}-{z}_{i}$ reflects the fidelity with which an ancestral character state is transmitted to descendant individuals. The change in the mean value of the character state is given by the Price equation:

4 $$\Delta \overline{z }={\sum }_{i}{q}_{i}^{^{\prime}}{z}_{i}^{^{\prime}}-\sum_{i}{q}_{i}{z}_{i}=\frac{1}{\overline{w} }\sum_{i}{q}_{i}{\left({w}_{i}-\overline{w }\right)z}_{i}+\frac{1}{\overline{w} }\sum_{i}{q}_{i}{w}_{i}\Delta {z}_{i}$$

The first sum on the RHS represents change due to selection. This term is non-zero whenever $\mathrm{var}\left({z}_{i}\right)>0$. The second sum accounts for change due to a combination of selection and transmission bias. This term is non-zero whenever $\Delta {z}_{i}\ne 0$. Note that fitness in the Price equation is not stochastic but represents realized values. The ancestor–descendant mapping is therefore deterministic. However, the mapping can be implemented stochastically via computer simulation.

Our Eq. 3 of the main paper was derived by substituting the expressions for transmission bias (Eq. 1) and fitness (Eq. 2) into the Price equation (Eq. 4):

$$\Delta \overline{z }=\frac{1}{\overline{w} }\sum_{i}{q}_{i}{\left({w}_{i}^{m}+{w}_{i}^{p}-\overline{w }\right)z}_{i}+\frac{1}{\overline{w} }\sum_{i}{q}_{i}\left({w}_{i}^{m}+{w}_{i}^{p}\right)\mathrm{E}\left(\Delta {z}_{i}\right)$$

Fitness due to multiplication ${w}_{i}^{m}$ can be dropped from the second sum because of the assumption that $\mathrm{E}\left(\Delta {z}_{i}\right)=0$ for descendant generated by HGT. Combining the two terms that account for fitness due to persistence ${w}_{i}^{p}$ gives our model equation (Eq. 3). The equation for the change in mean indispensability is like Eq. 3:

$$\Delta \overline{y }=\frac{1}{\overline{w}}{\sum }_{i}{q}_{i}\left({w}_{i}^{m}-\overline{w }\right){y}_{i}+\frac{1}{\overline{w}}{\sum }_{i}{q}_{i}{w}_{i}^{p}\left({y}_{i}+\mathrm{E}\left(\Delta {y}_{i}\right)\right)$$

Note, however, that the assumption that the indispensability of T is ${y}_{i}=0$ following HGT into a naïve microbial population (i.e., when T generates descendants by multiplication), makes the first summation zero. Hence, the change in the mean indispensability across all population of T is:

$$\Delta \overline{y }=\frac{1}{\overline{w}}{\sum }_{i}{q}_{i}{w}_{i}^{p}\left({y}_{i}+\mathrm{E}\left(\Delta {y}_{i}\right)\right)$$

This underscores the fact that the indispensability of T is a property of the relationship between T and its current microbial host population, which is maintained only so long as T persists in that host population.

## Abbreviations

CNE: Constructive neutral evolution
HGT: Horizontal gene transfer
STRING: Search tool for the retrieval of interacting genes/proteins
SIM1: Single-minded family transcription factor 1

## Footnotes

1 It is important to emphasize that, in the context of our model, a population of transferable genes can be construed as an individual unit with fitness. The $\left({y}_{1},{z}_{1}\right)$ variant is therefore analogous a wild type and the $\left({y}_{2},{z}_{2}\right)=\left({y}_{1},{z}_{1}-1\right)$ variant a mutant in the metapopulation of transferable genes.

2 Note that the itinerant life-history strategy of some genes is not in question here (e.g., [13]. What is at stake is the relationship between itinerance and the opportunity to colonization naïve microbial populations.

3 An alternative is to identify and measure an informative proxy for indispensability. One possibility is the rate of molecular evolution, which is predicted by neutral theory to be inversely related to functional constraint [24]. Indeed, Aris-Brosou [1] used this principle to show that genes whose products are highly connected tend to be more conserved and less likely to evolve by Darwinian positive selection.
