# Supplementary material for: Evolution of the connectivity and indispensability of a transferable gene: the simplicity hypothesis
Source: BMC Ecol Evol. 2022 Nov 30;22:140. doi: 10.1186/s12862-022-02091-w (PMC9710062; doi:10.1186/s12862-022-02091-w)
Supplement: Supplementary file 1 — Additional file 1. Deterministic calculations (Fig. 2). Stochastic simulations (Fig. 3) [file 12862_2022_2091_MOESM1_ESM.docx]

**Transitions in the state of a transferable gene by gene-host coevolution: the simplicity hypothesis.**

Jones, C. T., Postdoctoral Research Fellow, Dept. Biochemistry and Molecular Biology, Dalhousie University, Halifax, Nova Scotia [cjones2@dal.ca](mailto:cjones2@dal.ca), <https://orcid.org/0000-0002-2098-9771>

Susko, E., Dalhousie University, Dept. of Mathematics and Statistics

Bielawski, J. P., Dalhousie University, Dept. of Biology and Dept. of Mathematics and Statistics

**Additional file 1**

The document contains pseudocode for the deterministic calculations and stochastic simulations reported in the main paper. All simulations and calculations were implemented in MATLAB version R2021a under license number 861043 for academic use using custom scripts. Scripts are available on GitHub, <https://doi.org/10.5281/zenodo.7194737>

**Deterministic Calculations (Fig. 2)**

**Set parameters:**

- Number of Populations of T: ${N=10}^{3}$
- Initial Character State: $\left( y,z \right)=\left( 10,10 \right)$
- Probability of a shift to the Neutral Environment: $\delta=0.01$
- Maximum Size of the Metapopulation: $N_{max}={10}^{4}$
- Scaling Constant: $s=0.20$
- Number of Mappings: ${10}^{4}$

**Initialize Population Numbers** $\boldsymbol{n(y,z,t)}$ **and Proportions** $\boldsymbol{q(y,z,t)}$**:**

- Time $t$ represents the number of ancestor-descendant mappings, starting at $t=1$.
- $n\left( 10,10,1 \right)={10}^{3}$, the initial size of the metapopulation with $\left( y,z \right)=\left( 10,10 \right)$
- $q\left( 10,10,1 \right)=1$
- $n\left( 0,10,1 \right)=0$, new populations generated by HGT with $\left( y,z \right)=\left( 0,10 \right)$
- $q\left( 0,10,1 \right)=0$

**For each ancestor-descendant mapping:**

Calculate the components of fitness $w^{p}\left( y \right)$ and $w^{m}(z,t)$:

- $w^{p}\left( 0 \right)=1-\delta$
- $w^{p}\left( 10 \right)=1-\delta\exp\left( -10s \right)$
- $w^{m}(10,t)= \beta\left( 1-N(t){/N}_{max} \right)\exp\left( -10s \right)$
- where $N(t)=n\left( 10,10,t \right)+n\left( 0,10,t \right)$ is the number of populations of X at time $t$

Calculate Mean Fitness:

- $\bar{w}= q\left( 10,10,t \right)\times\left( w^{p}\left( 10 \right)+w^{m}\left( 10,t \right) \right)+q\left( 0,10,t \right)\times\left( w^{p}\left( 0 \right)+w^{m}\left( 10,t \right) \right)$

Update Population Numbers (without rounding to the nearest integer):

- $n\left( 10,10,t+1 \right)=n\left( 10,10,t \right)\times w^{p}\left( 10 \right)$
- $n\left( 0,10,t+1 \right)=n\left( 10,10,t \right)\times w^{m}\left( 10,t \right)+ n\left( 0,10,t \right)\times\left( w^{p}\left( 0 \right)+w^{m}\left( 10,t \right) \right)$

Update Proportions:

- $q\left( 10,10,t+1 \right)=q\left( 10,10,t \right)\times w^{p}\left( 10 \right)/\bar{w}$
- $q\left( 0,10,t+1 \right)=q\left( 10,10,t \right)\times w^{m}\left( 10,t \right)/\bar{w}+q\left( 0,10,t \right)\times\left( w^{p}\left( 0 \right)+w^{m}\left( 10,t \right) \right)/\bar{w}$

**Stochastic Simulations (Figs. 3)**

**Set parameters:**

- Number of Populations of T: ${N=10}^{3}$
- Initial Character State: $\left( y,z \right)=\left( 10,10 \right)$
- Probability of a shift to the Neutral Environment: $\delta=0.01$
- Maximum Size of the Meta Population: $N_{max}={10}^{4}$
- Scaling Constant: $s=0.20$
- Number of Mappings: ${10}^{4}$
- $P\left( -1 \right)={10}^{-5}, P\left( +1 \right)={10}^{-3}$, $P\left( 0 \right)=1-P\left( -1 \right)-P(+1)$

**Initialize Population Numbers** $\boldsymbol{n(y,z,t)}$ **and Proportions** $\boldsymbol{q(y,z,t)}$**:**

- Time $t$ represents the number of ancestor-descendant mappings, starting at $t=1$.
- $n\left( 10,10,1 \right)={10}^{3}$, the initial size of the metapopulation with $\left( y,z \right)=\left( 10,10 \right)$
- $q\left( 10,10,1 \right)=1$

**For each ancestor-descendant mapping:**

Account for births by HGT:

- For each ancestral population of T that exists at time $t$, draw a Poisson random variable $V$ with expected value $E\left( V \right)= \beta\left( 1-N(t){/N}_{max} \right)$, where $N(t)$ is the total number of populations of T at time $t$. $V$ represents the number of naïve microbial populations an ancestral population of T will enter by HGT over the next ancestral-descendant mapping.
- For each naïve population into which T with $\left( y_{i},z_{i} \right)$ entered by HGT, draw a Bernoulli random variable $B$ with expected value $E\left( B \right)=\exp\left( -z_{i}\times s \right)$. This is the probability that T is fixed in that microbial population.
- A descendant population of T with $\left( 0,z_{i} \right)$ is generated whenever $B=1$.

Account for deaths by gene loss:

- For each ancestral population of T at time $t$, draw a Bernoulli random variable $D$ with expected value $E\left( D \right)=\delta\exp\left( -y_{i}\times s \right)$.
- The ancestral population of T with $\left( y_{i},z_{i} \right)$ suffers death by gene loss whenever $D=1$ but persists whenever $D=0$.

Account for CNE for each ancestral population of T with $\left( y_{i},z_{i} \right)$ that persists:

- If $y_{i}=0$, then draw a random variable $M$ from a Bernoulli distribution with expected value $E\left( M \right)=P(+1)/\left( P\left( 0 \right)+P\left( +1 \right) \right)$. If $M=1$, then change the character state of T from $\left( 0,z_{i} \right)$ to $\left( 1,z_{i} \right)$.
- If $y_{i}\geq1$, then draw a random variable $M$ from a multinomial distribution with vector of probabilities $\pi=\left\langle P\left( -1 \right),P(0), P(+1) \right\rangle$. If $M=\left\langle1,0,0 \right\rangle$ or $M=\left\langle0,0,1 \right\rangle$, then change the character state of X from $\left( y_{i},z_{i} \right)$ to $\left( y_{i}-1,z_{i} \right)$ or $\left( y_{i}+1,z_{i} \right)$, respectively.
- If $z_{i}=0$, then draw a random variable $M$ from a Bernoulli distribution with expected value $E\left( M \right)=P(+1)/\left( P\left( 0 \right)+P\left( +1 \right) \right)$. If $M=1$, then change the character state of T from $\left( y_{i},0 \right)$ to $\left( y_{i},1 \right)$.
- If $z_{i}\geq1$, then draw a random variable $M$ from a multinomial distribution with vector of probabilities $\pi=\left\langle P\left( -1 \right),P(0), P(+1) \right\rangle$. If $M=\left\langle1,0,0 \right\rangle$ or $M=\left\langle0,0,1 \right\rangle$, then change the character state of X from $\left( y_{i},z_{i} \right)$ to $\left( y_{i},z_{i}-1 \right)$ or $\left( y_{i},z_{i}+1 \right)$, respectively.

Bookkeeping:

- Keep track of the number of all variants of T with that exist at time $t+1$ after accounting for births, deaths, and change due to CNE.
